# Selective formation of a zwitterion adduct and bicarbonate salt in the efficient CO_2_ fixation by *N*-benzyl cyclic guanidine under dry and wet conditions

**DOI:** 10.3762/bjoc.14.194

**Published:** 2018-08-23

**Authors:** Yoshiaki Yoshida, Naoto Aoyagi, Takeshi Endo

**Affiliations:** 1 Molecular Engineering Institute, Kindai University, 11-6 Kayanomori, Iizuka, Fukuoka 820-8555, Japan

**Keywords:** bicarbonate salt, carbon dioxide adsorption, cyclic guanidine, repeatable capture and release, zwitterion adduct

## Abstract

The efficient CO_2_ fixation by *N*-benzyl cyclic guanidine **1** was achieved by bubbling dry CO_2_ through CH_3_CN at 25 °C for 2 h. In addition, the zwitterion adduct **2** and bicarbonate salt **3** were selectively prepared from **1** under dry (in anhydrous CH_3_CN) and wet (in CH_3_CN containing an equimolar amount of water for **1**) conditions, respectively. Both compounds **2** and **3** were isolated as white solids and their structures were characterized in detail by elemental analysis, FTIR-ATR, solid-state NMR, TGA, and DFT calculation. These analytical results obviously revealed the formation of a zwitterion adduct and bicarbonate salt from *N*-benzyl cyclic guanidine and CO_2_. Especially, the zwitterion adduct of the monocyclic guanidine derivative and CO_2_ was isolated and characterized for the first time.

## Introduction

Recently, various reactions with CO_2_ as a cheap and green carbon reagent have been developed not only in the field of organic and catalyst chemistry but also in inorganic and polymer chemistry. Especially the adsorbent and the catalyst for CO_2_ fixation are of interest [1–5]. In particular, the inorganic, organic–inorganic hybrid, dendrimeric catalysts have been developed for chemical fixation of CO_2_ such as cycloaddition of CO_2_ to various epoxides and synthesis of carbonate derivatives from CO_2_ and alcohols [6–13]. However, these kinds of catalysts often need high temperatures and pressure to completely achieve the CO_2_ fixation [6]. On the other hand, amidine and guanidine derivatives are well known as efficient catalysts for the cycloaddition of CO_2_ to epoxides at ambient temperature [7,14–18]. Previously, we reported that hydroiodides of amidine derivatives worked as significantly efficient catalysts for the cycloaddition of CO_2_ to epoxides under ambient temperature and pressure [19–22]. Furthermore, cyclic amidines and guanidines, such as 1,8-diazabicyclo[5.4.0]undec-7-ene (DBU) and 1,5,7-triazabicyclo[4.4.0]dec-5-ene (TBD), exhibited an excellent efficiency of CO_2_ capture and release [23–33]. In particular, CO_2_ capture–release behaviors of bicyclic and six-membered cyclic amidine derivatives have been well studied compared to that of five-membered derivatives, because the high ring strain of five-membered cyclic amidine derivatives was unfavorable for the binding between CO_2_ and the amidine moiety [34–38]. However, we found that five-membered cyclic guanidine was excellently efficient for CO_2_ capture under dry conditions because the trapped CO_2_ was significantly stabilized as the bicarbonate together with a slight amount of water due to the specific basicity based on the resonance structure of the guanidine moiety [39]. Furthermore, we also have demonstrated that the adsorption performance of CO_2_ was fairly different under dry and wet conditions [35]. Previously, Jessop et al. described that the bicarbonate salt of DBU and CO_2_ was obtained in the presence of water and never confirmed in the absence of water [23]. Moreover, Pérez et al. indicated that the DBU–CO_2_ complex was assigned as the zwitterion adduct of DBU and CO_2_ involving water by ^13^C NMR, TGA, and elemental analysis, although the bicarbonate salt was formed due to hydrolysis in the DBU–CO_2_ carbamic complex involving water during the crystallization process [28]. On the other hand, Villiers and Ephritikhine et al. synthesized and characterized successfully the zwitterion adduct of CO_2_ and TBD under strictly anhydrous conditions [31]. They also described that the obtained zwitterion adduct of TBD–CO_2_ was stable at room temperature in a dry atmosphere of argon for at least one month. This report is a limited example on the isolated zwitterion adduct of guanidine derivatives and CO_2_. As such, several researchers have demonstrated about the formation of zwitterion adduct and/or bicarbonate salt between guanidine derivatives and CO_2_ under dry and/or wet conditions. Previously, there were a lot of reports about the bicarbonate salt of guanidine derivatives and CO_2_ [23–40]. On the other hand, preparation and isolation of the zwitterion adduct have been barely reported, because the zwitterion adduct was readily transformed to the bicarbonate salt owing to ambient moisture. Therefore, it is worthwhile to synthesize and isolate the moisture stable zwitterionic adduct of guanidine derivatives and CO_2_, because the zwitterionic adduct contains an activated carbon dioxide without a water molecule. It is expected that the zwitterion adducts of guanidine derivatives and CO_2_ are not only efficient catalysts for cycloaddition of CO_2_ to epoxides but also a thermal latent curing agent for epoxy resin. Herein, we achieved selective formation of the zwitterionic adduct and bicarbonate salt between CO_2_ and *N*-benzyl cyclic guanidine **1**. The structures of the zwitterionic adduct and bicarbonate salt were analyzed in detail and proved by elemental analysis, FTIR-ATR, solid-state NMR, TGA, and DFT calculations. In this report, the zwitterionic adduct of the monocyclic guanidine derivative and CO_2_ was isolated and characterized for the first time, although there were a few reports about the bicyclic guanidine derivatives such as TBD.

## Results and Discussion

### CO_2_ fixation behavior of *N*-benzyl cyclic guanidine depending on dry and wet conditions

**Scheme 1:**
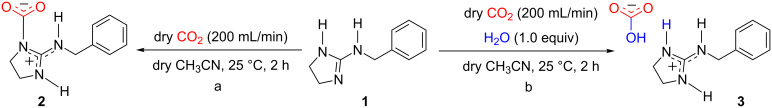
Fixation of CO_2_ (200 mL/min) by **1** under (a) dry and (b) wet conditions.

**Figure 1:**
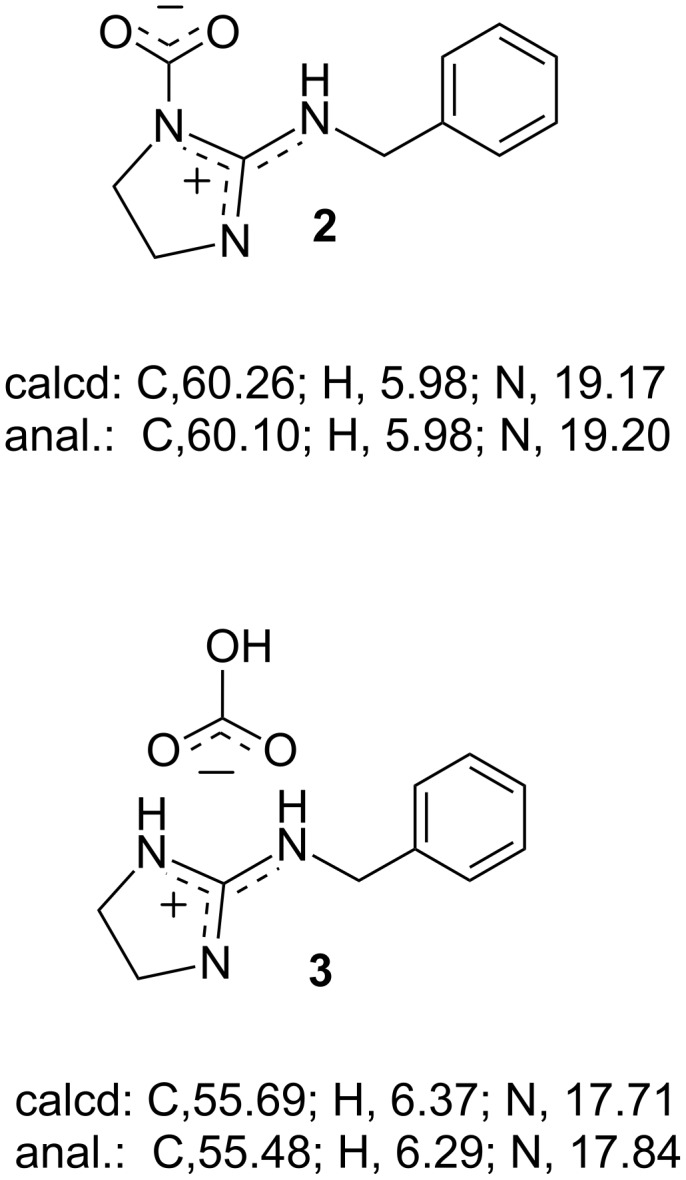
Zwitterion adduct **2** and bicarbonate salt **3** confirmed by elemental analysis.

**Figure 2:**
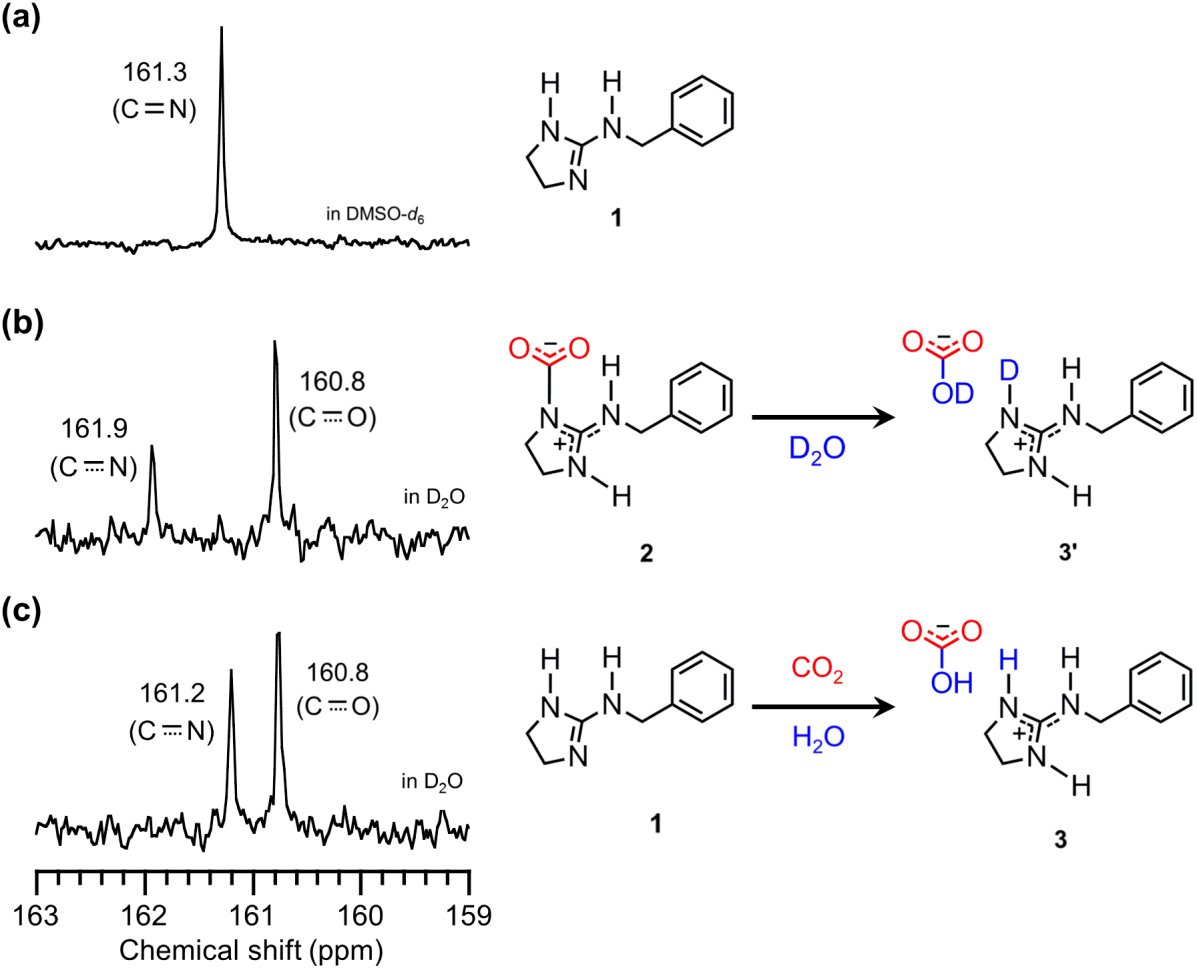
^13^C NMR spectra of (a) **1** observed in DMSO-*d*_6_, (b) **3'** prepared with **2** and D_2_O observed in D_2_O, and (c) **3** prepared with **1** under wet conditions observed in D_2_O.

First, CO_2_ fixation by *N*-benzyl cyclic guanidine **1** was carried out by bubbling dry CO_2_ through anhydrous CH_3_CN unter dry conditions (Scheme 1a) and through CH_3_CN containing an equimolar amount of water for **1** as wet condition (Scheme 1b) at 25 °C for 2 h. The white solid was immediately precipitated in the homogeneous solution of **1** during CO_2_ bubbling under both conditions. The efficient absorption of CO_2_ by **1** suggested that the trapped CO_2_ was stabilized by the active hydrogen on the nitrogen atom. This hydrogen was fairly activated due to the resonance effect of the guanidine moiety [16,39]. The compositional formula of the obtained white solids was confirmed by elemental analysis. This result predicted that the zwitterionic adduct **2** and the bicarbonate salt **3** were prepared selectively under dry and wet conditions, respectively (Figure 1). Interestingly, **3** was barely obtained by the addition of an equivalent water to the precipitation of **2** in CH_3_CN, and then the only part of **2** was transformed to **3** after stirring in CH_3_CN containing an excess mole of water, although **2** and **3** were insoluble in organic solvents. This result means that **2** is hydrophobic enough to be isolated as an individual compound. On the other hand, **3** was also prepared from **2** dissolved completely in water, because ^1^H NMR spectra of **2** and **3** showed the same signals in D_2_O (Supporting Information File 1, section 2-1) and the carbonyl peaks due to the bicarbonate moiety were observed at 160.8 ppm in D_2_O by ^13^C NMR spectra of **2** and **3** (Figure 2).

### Characterization of zwitterion adduct **2** and bicarbonate salt **3** by FTIR-ATR and solid-state ^13^C-CPMAS NMR

**Figure 3:**
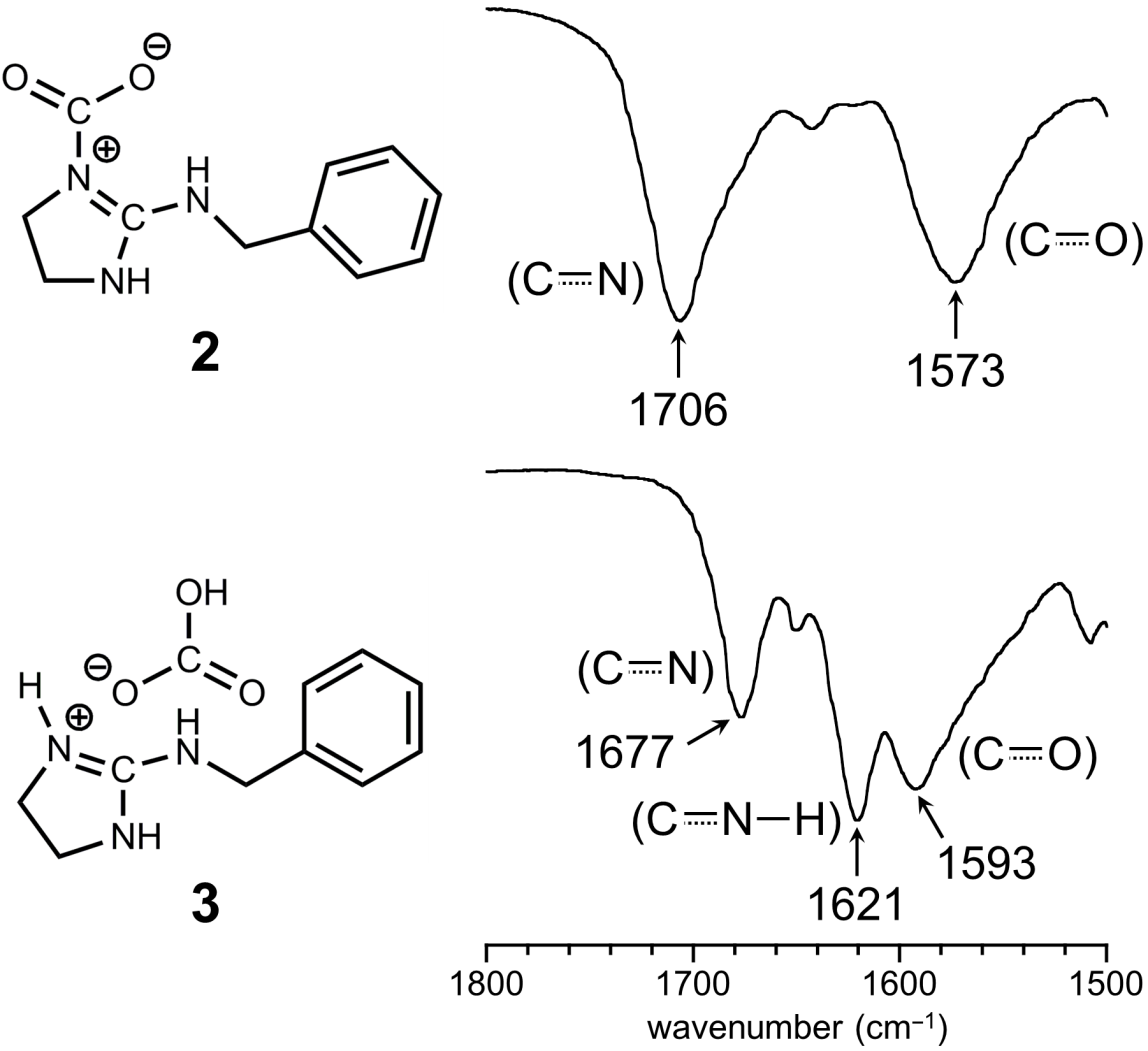
FTIR-ATR spectra of zwitterion adduct **2** and bicarbonate salt **3** expanded at the range of 1500–1800 cm^−1^.

IR spectra of **2** and **3** were measured by FTIR-ATR methods (Figure 3). The two peaks were clearly observed at 1573 cm^−1^ and 1706 cm^−1^ due to carbonyl and imine moieties, respectively, in the spectrum of **2**. On the other hand, the peak at 1593 cm^−1^ was assigned to the carbonyl group of the bicarbonate moiety, and also the peaks due to C=N and N–H of iminium group in guanidinium moiety were observed at 1677 cm^−1^ and 1621 cm^−1^, respectively, in the spectrum of **3**.

**Figure 4:**
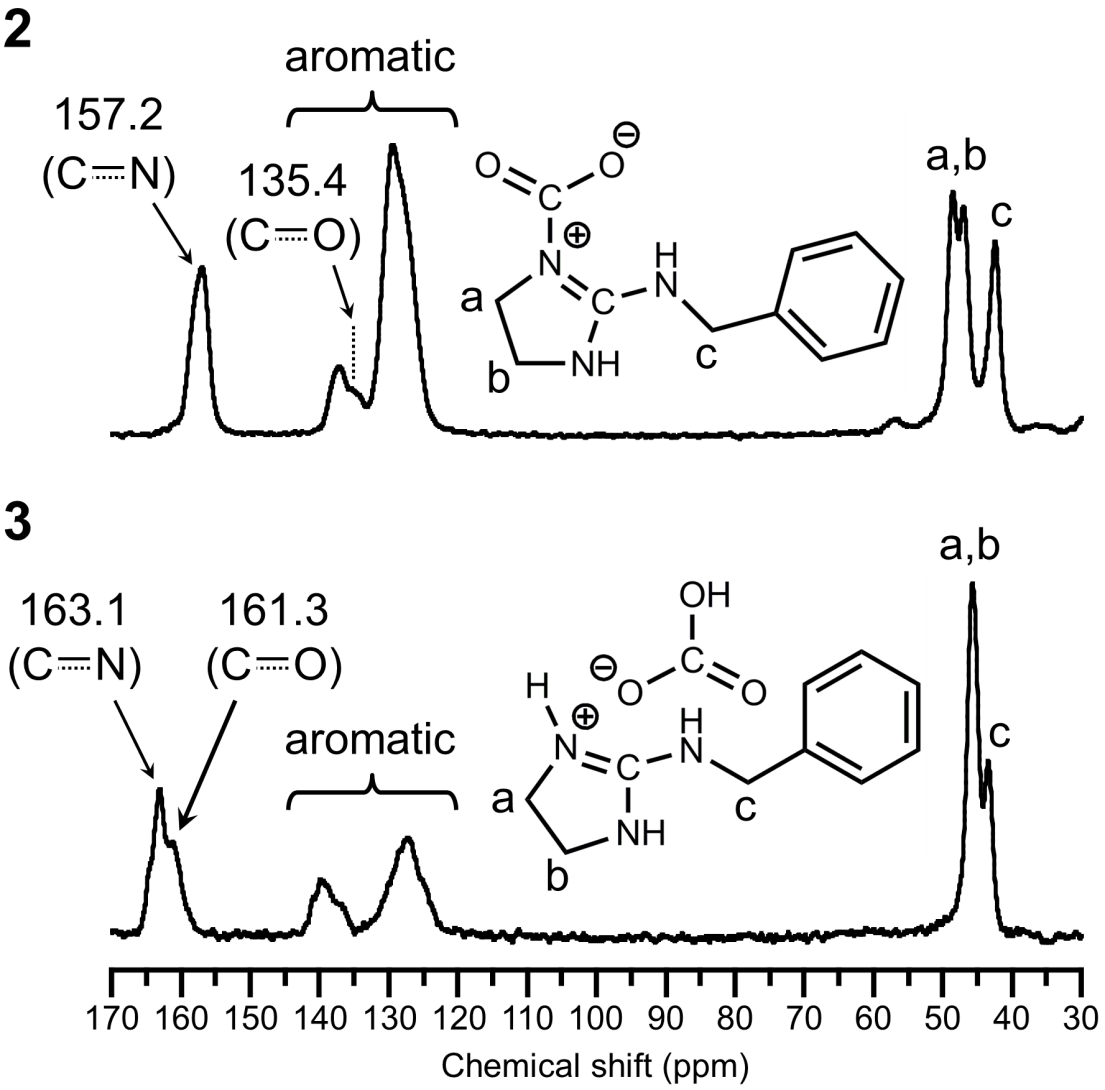
^13^C-CPMAS NMR spectra of zwitterion adduct **2** and bicarbonate salt **3** expanded at the range of 30–170 ppm.

Moreover, the structures of **2** and **3** were also revealed by ^13^C-CPMAS NMR in solid-state (Figure 4). The carbon peaks due to the trapped CO_2_ and guanidinium moieties were observed at 135.4 ppm and 157.2 ppm, respectively, in the spectrum of **2**, which indicated the zwitterionic structure between CO_2_ and **1**. On the other hand, the carbon peaks due to the bicarbonate and guanidinium moieties were observed at 161.3 ppm and 163.1 ppm, respectively, in the spectrum of **3**. This chemical shift of **2** suggested that the electron density on the carbonyl carbon of **2** increased by the donation of an electron from the imine nitrogen, and also the electron at the iminium carbon was localized due to the imine structure stabilized by the direct binding with CO_2_. Therefore, both the peaks of carbonyl and iminium carbons in **2** shifted to higher magnetic field compared to that of **3**. On the other hand, both the peaks of bicarbonate and guanidinium carbons in **3** were observed at a low magnetic field, because the electrons on bicarbonate and guanidinium carbons were delocalized on oxygen and nitrogen atoms by their resonance effect, respectively. Previously, some researchers have reported similar assignments for zwitterion adducts of amidines and guanidines [29–31]. The IR and NMR spectra indicated that **2** and **3** were selectively prepared under dry and wet conditions, respectively, and then **2** and **3** were obtained as fairly stable compounds under ambient temperature and moisture.

### Computational evaluation for the CO_2_ fixation behavior of *N*-benzyl cyclic guanidine

**Figure 5:**
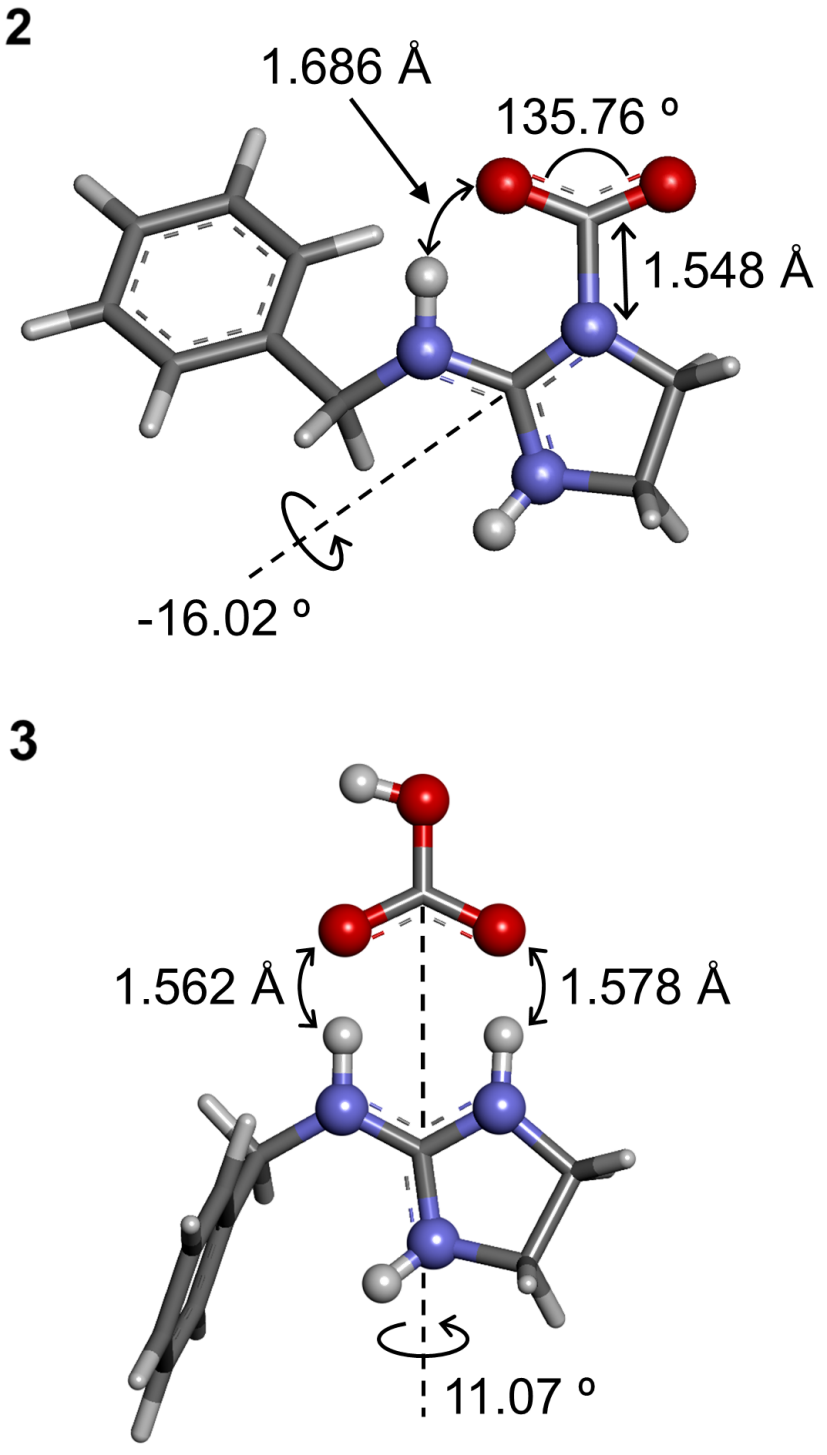
The optimized geometries of zwitterion adduct **2** and bicarbonate salt **3** estimated by DFT calculation (B3LYP/6-31G*).

The geometrical structures of **2** and **3** were computed with DFT calculation (Figure 5). In the calculated geometry for **2**, the bond length between CO_2_ and imine nitrogen was 1.548 Å and the bent angle of O–C–O bond was 135.76°. These bond length and angle were fairly similar to that of the zwitterion adduct between CO_2_ and TBD calculated by Wei and Sun et al. [40]. Moreover, the distance between the trapped CO_2_ and the active hydrogen at the nitrogen atom was close to each other (1.686 Å), which also agreed with that of TBD–CO_2_ adduct calculated by Villiers and Ephritikhine et al. [31]. On the other hand, the calculated geometry for **3** showed that the distance between the two protons of the imidazolinium moiety and two oxygen atoms of the bicarbonate moiety were fairly close to each other (1.562 Å and 1.578 Å), indicating that the two oxygen atoms on the bicarbonate are stabilized by the active hydrogen on the imidazolinium moiety. Furthermore, due to the guanidine moiety, three nitrogen atoms of **2** and **3** were in a more planar position than that of **1**, which was the twisted conformation between the benzylamino group and the cyclic amidine moiety (Supporting Information File 1, section 1-1). Therefore, these results strongly supported the geometrical structures and electronic state of **2** and **3** predicted by ^13^C NMR and IR spectra.

### Thermal decomposition behaviors of zwitterion adduct **2** and bicarbonate salt **3**

**Figure 6:**
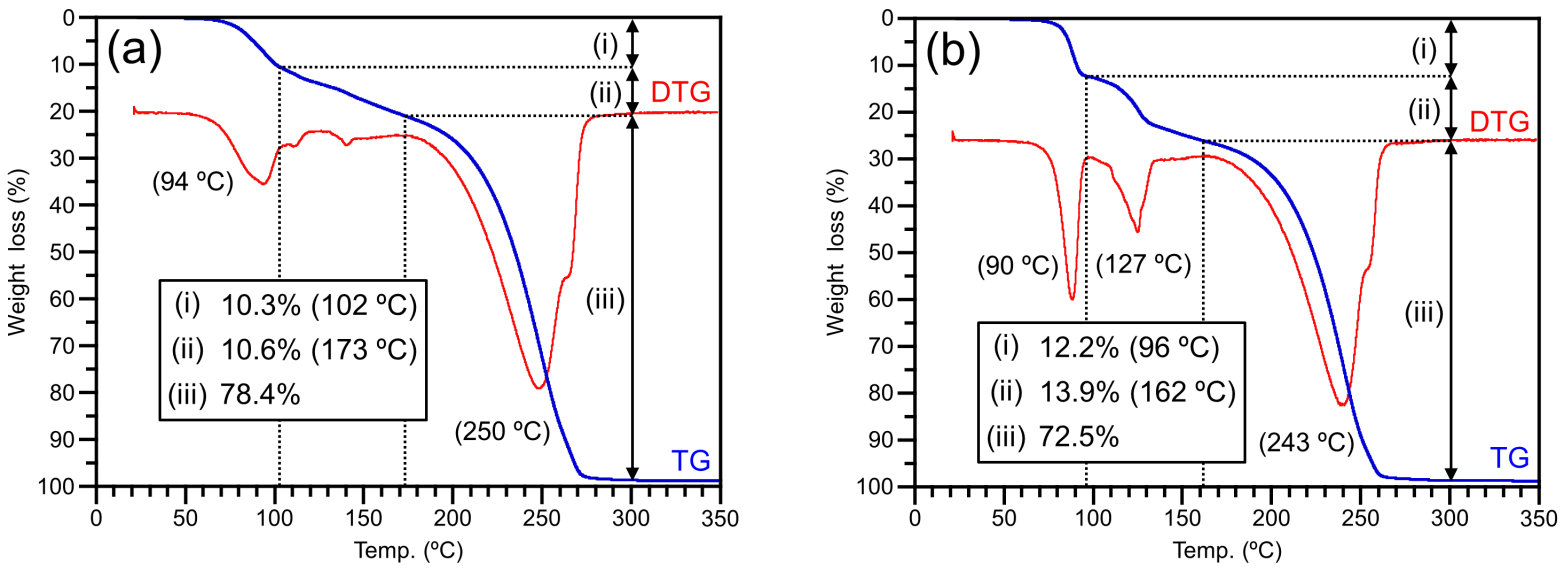
TGA trace of (a) zwitterion adduct **2** and (b) bicarbonate salt **3** observed under N_2_ flow (200 mL/min) at heating rate of 5 °C/min.

**Scheme 2:**
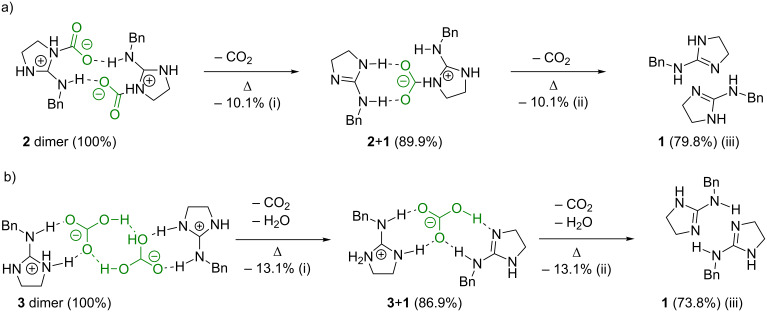
Proposal decomposition paths and theoretical weight loss values of (a) zwitterion adduct **2** and (b) bicarbonate salt **3**.

Thermal decomposition behaviors of **2** and **3** were observed by TGA measurement. TGA traces and weight loss values of **2** and **3** are shown in Figure 6. Moreover, their proposed decomposition paths and the theoretical values of weight loss are shown in Scheme 2. The weight loss of **2** exhibited a two-step behavior before complete thermal decomposition (Figure 6a). Both the first and second weight loss proceeded slowly until 102 °C (i) and 173 °C (ii), respectively, and then the thermal decomposition of **2** has occurred completely around 270 °C (iii). This three-step behavior of weight loss suggested the dimer form of **2**, which was constructed by the weak interaction between the anionic oxygen on the trapped CO_2_ and the active hydrogen on the nitrogen atom. Namely, one molecule of CO_2_ was released from the dimer of **2** in the first step, and then the intermediate complex was prepared from **1** and **2** due to the coordination between two anionic oxygens on the trapped CO_2_ and two active hydrogens on the guanidine moiety. Then, another CO_2_ molecule was released from the intermediate complex (**2**+**1** dimer) and the dimer was decomposed into two molecules of **1** in the second step. The observed weight loss due to the released CO_2_ in each step agreed with the theoretical one (obs. (i) 10.3%, (ii) 10.6%, and (iii) 78.4%, theor. (i) 10.1%, (ii) 10.1%, and (iii) 79.8%). On the other hand, the weight loss of **3** also exhibited a three-step behavior as well as **2**, although the TGA trace pattern was perfectly different from that of **2** (Figure 6b). The first weight loss occurred rapidly around 90 °C (until 96 °C) (i), and then the second weight loss exhibited rapid and slow weight loss behaviors around 127 °C and until 162 °C (ii), respectively. Finally, the thermal decomposition of **3** has occurred completely around 260 °C (iii). This three-steps behavior of weight loss suggested that the dimer of **3** was constructed by the two hydrogen bonds between two oxygen atoms in two HCO_3_^−^ anions. Recently, we demonstrated that the bicarbonate salt of the cyclic guanidine derivative was decomposed at three-steps [39]. Similarly, the first weight loss until 96 °C was assigned to the elimination of one pair of CO_2_ and H_2_O due to the bicarbonate moiety in the dimer of **3**. Accordingly, the intermediate complex of **3** and **1** was constructed by the two hydrogen bonds between O**H**−**N** and **O**−**H**N in the bicarbonate salt **3** and the free guanidine **1**, and then another one pair of CO_2_ and H_2_O was almost released from the intermediate complex (**3**+**1** dimer) around 127 °C in the second step. After the rapid elimination of CO_2_ and H_2_O around 127 °C, the remained bicarbonate moiety was also released slowly from **3**+**1** dimer until 162 °C, because the bicarbonate moiety was probably stabilized by a large amount of the free guanidine. The observed weight loss from the dimer of **3** also agreed with the theoretical one as well as **2** (obs. (i) 12.2%, (ii) 13.9%, and (iii) 72.5%, theor. (i) 13.1%, (ii) 13.1%, and (iii) 73.8%). Therefore, this thermal decomposition behavior indicated that the dimers of zwitterion adduct and bicarbonate salt were constructed in solid-state and their structure was fairly stabilized because of the multi hydrogen bond in the intermolecular.

## Conclusion

The efficient CO_2_ fixation by *N*-benzyl cyclic guanidine **1** was achieved by bubbling dry CO_2_ in CH_3_CN at 25 °C for 2 h. In addition, the zwitterion adduct **2** and bicarbonate salt **3** were selectively prepared from **1** under dry (in anhydrous CH_3_CN) and wet (in CH_3_CN containing an equimolar amount of water for **1**) conditions, respectively. Both of **2** and **3** were isolated as white solids and their structures were characterized in detail by elemental analysis, FTIR-ATR, solid-state NMR, TGA, and DFT calculation. Especially, the results of the thermal analysis revealed that the obtained zwitterion adduct and bicarbonate salt stabilized the dimer complexes based on multiple intermolecular hydrogen bonds. This also indicated that the zwitterion adduct, bicarbonate salt, and their dimers were a fairly stable structure at room temperature and ambient moisture.

## Experimental

### General

#### Materials and instruments

All starting materials and dehydrated solvents were purchased from Wako Pure Chemical Industries (Osaka, Japan) and Tokyo Chemical Industry (Tokyo, Japan). CO_2_ (>99.999%, H_2_O < 5 ppm) was obtained from Yamagata Sanso (Yamagata, Japan). The NMR spectra were obtained using a JEOL ECS-400 spectrometer operating at 400 MHz for ^1^H and 100 MHz for ^13^C. The elemental analyses were performed by a Perkin Elmer 2400II CHNS/O Analyzer. Mass spectroscopy was performed on a Shimadzu GCMS-QP2010SE in electron ionization (EI) mode. FTIR spectra were recorded on a Thermo Scientific Nicolet iS10 spectrometer equipped with a Smart iTR diamond ATR sampling accessory in the range of 4000–650 cm^−1^. Solid-state NMR, ^13^C cross-polarization (CP)/magic angle spinning (MAS, 99.5 MHz) measurements were performed on a JEOL JNM-ECX 400 spectrometer, at a spinning speed of 10 kHz. Thermogravimetric analysis (TGA) was performed on a Seiko Instrument TG-DTA 6200 using an aluminum pan in the temperature range of 30–350 °C at a heating rate of 5 °C/min under a nitrogen atmosphere (flow rate 200 mL/min). Geometry optimized energy for **1**, **2** and **3** was estimated by using DFT calculation with B3LYP/6-31G* method (Wavefunction, Inc., Spartan’06 Windows version 1.1.0) [41].

#### Synthesis of *N*-benzyl cyclic guanidine

2-Benzylamino-4,5-dihydro-1*H*-imidazole (*N*-benzyl cyclic guanidine, **1**) was synthesized according to the literature [42]. mp 79.9–83.1 °C; ^1^H NMR (400 MHz, DMSO-*d*_6_, 25 °C) δ (ppm) 7.20–7.33 (m, 7H), 4.26 (s, 2H), 3.37 (s, 4H); ^13^C NMR (100 MHz, DMSO-*d*_6_, 25 °C) δ (ppm) 161.3, 140.3, 128.0, 127.1, 126.5, 46.5, 46.0; EIMS (*m*/*z*): 175 (M^+^).

#### Procedure for CO_2_ fixation under dry and wet conditions [39]

Dry CO_2_ gas (>99.999%, H_2_O <5 ppm) was bubbled into a solution of **1** (4 mmol) in anhydrous CH_3_CN (10 mL) at a flow rate of 200 mL/min at 25 ºC. After 2 h under bubbling CO_2_, the resulting white precipitate was filtered off, washed with anhydrous Et_2_O (10 mL × 3) and dried in a stream of CO_2_ (at a flow rate of 200 mL/min at 25 °C for 4 h) to give zwitterion adduct **2** as a white powder.

**Zwitterion adduct 2:** Yield 798 mg, 91%; mp 92.1–109.4 °C; IR (ATR, cm^−1^) 1706 (C=N), 1573 (C=O); ^13^C-CPMAS NMR (99.5 MHz, 25 °C) δ (ppm) 157.2 (*C*=N), 137.3 (aromatic), 135.4 (*C*O_2_), 129.5 (aromatic), 48.6 and 47.1 (N*C*H_2_*C*H_2_NH), 42.6 (*C*H_2_Ph); anal. calcd for C_11_H_13_N_3_O_2_: C, 60.26; H, 5.98; N, 19.17; found: C, 60.10; H, 5.98; N, 19.20.

#### CO_2_ fixation of **1** under wet conditions

Dry CO_2_ gas (>99.999%, H_2_O <5 ppm) was bubbled into a solution of **1** (4 mmol) in CH_3_CN (10 mL) containing water (0.1 mL) at a flow rate of 200 mL/min at 25 ºC. After 2 h under bubbling CO_2_, the resulting white precipitate was filtered off, washed with anhydrous Et_2_O (10 mL × 3) and dried in a stream of CO_2_ (at a flow rate of 200 mL/min at 25 ºC for 4 h) to give bicarbonate salt **3** as a white powder.

**Bicarbonate salt 3:** Yield 930 mg, 98%; mp 89.7–109.2 °C; IR (ATR, cm^−1^) 1677 (C=N), 1621 (C=N−H), 1593 (C=O); ^13^C-CPMAS NMR (99.5 MHz, 25 °C) δ (ppm) 163.1 (*C*=N), 161.3 (H*C*O_3_), 139.8 and 127.7 (aromatic), 45.7 (N*C*H_2_*C*H_2_NH), 43.5 (*C*H_2_Ph); anal. calcd for C_11_H_15_N_3_O_3_: C, 55.69; H, 6.37; N, 17.71; found: C, 55.48; H, 6.29; N, 17.84.

## Supporting Information

File 1 DFT computational results, FTIR-ATR, and NMR spectra of **1**, **2** and **3**.
