# Supplementary material for: Selective formation of a zwitterion adduct and bicarbonate salt in the efficient CO2 fixation by N-benzyl cyclic guanidine under dry and wet conditions
Source: Beilstein J Org Chem. 2018 Aug 23;14:2204–11. doi: 10.3762/bjoc.14.194 (PMC6122324; doi:10.3762/bjoc.14.194)

Supporting Information

for

Selective formation of a zwitterion adduct and bicarbonate salt in the efficient CO_2 fixation by *N*-benzyl cyclic guanidine under dry and wet conditions

Yoshiaki Yoshida, Naoto Aoyagi and Takeshi Endo*

Address: Molecular Engineering Institute, Kindai University, 11-6 Kayanomori, Iizuka, Fukuoka 820-8555, Japan

Email: Takeshi Endo - tendo@moleng.fuk.kindai.ac.jp

*Corresponding author

DFT computational results, FTIR-ATR, and NMR spectra of 1, 2 and 3

Table of contents

1. Energy and Cartesian Coordinates of the Optimized Geometries
1-1. For **1**, S2
1-2. For **2**, S5
1-3. For **3**, S9
2. Spectral copies of FTIR-ATR and solid-state ^{13}C-CPMAS NMR data
2-1. ^{1}H NMR spectra of (a) **1** observed in DMSO-d_6, (b) **3'** prepared with **2** and D_2O observed in D_2O, and (c) **3** prepared with **1** under wet condition observed in D_2O, S12
2-2. ^{13}C NMR spectra of (a) **1** observed in DMSO-d_6, (b) **3'** prepared with **2** and D_2O observed in D_2O, and (c) **3** prepared with **1** under wet condition observed in D_2O, S13
2-3. FTIR-ATR spectrum of **1**, S14
2-4. (a) FTIR-ATR and (b) ^{13}C-CPMAS NMR spectra of **2**, S15
2-5. (a) FTIR-ATR and (b) ^{13}C-CPMAS NMR spectra of **3**, S16

1-1 Energy and Cartesian Coordinates of the Optimized Geometries for 1

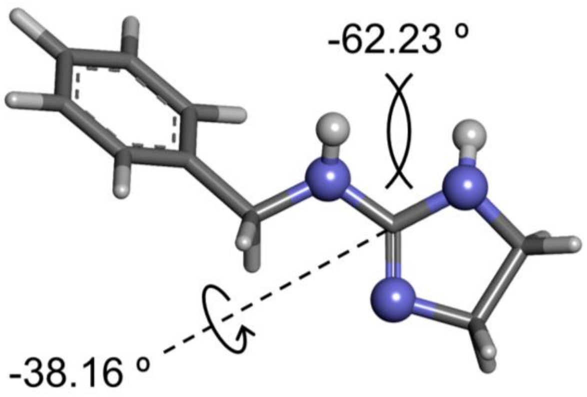

SPARTAN 'O6 Mechanics Program: (PC/x86) Release 127

Reading coordinates from previous archive
Adjusted 2 (out of 78) low frequency modes

Reason for exit: Successful completion
Mechanics CPU Time : 000:00:00.2

SPARTAN 'O6 Quantum Mechanics Program: (PC/x86) Release 127v3

Job type: Geometry optimization.
Method: RB3LYP
Basis set: 6-31G(D)
Number of shells: 78
Number of basis functions: 221

SCF model:
A restricted hybrid HF-DFT SCF calculation will be
performed using Pulay DIIS + Geometric Direct Minimization

Optimization:

Step	Energy	Max Grad.	Max Dist.
1	-553.1304065	0.040280	0.104876
2	-553.1363930	0.006263	0.056872
3	-553.1367533	0.001213	0.003240
4	-553.1367635	0.001201	0.003240

5	-553.1367725	0.001186	0.003240
6	-553.1367811	0.001172	0.003240
7	-553.1367895	0.001158	0.003240
8	-553.1367979	0.001143	0.003240
9	-553.1368062	0.001129	0.003240
10	-553.1368127	0.001116	0.118995
11	-553.1370493	0.000675	0.127202
12	-553.1371394	0.000544	0.065770
13	-553.1371661	0.000452	0.129013
14	-553.1372056	0.000551	0.025809
15	-553.1372085	0.000272	0.018186
16	-553.1372098	0.000144	0.005555
17	-553.1372110	0.000060	0.003337
18	-553.1372111	0.000035	0.001155

Program Wall Time: 2:02:06.0

Reason for exit: Successful completion
Quantum Calculation CPU Time : 001:18:11.4

SPARTAN 'O6 Semi-Empirical Program: (PC/x86) Release 127
Semi-empirical Property Calculation

M001

Guess from Archive

Energy Due to Solvation
Solvation Energy SM5.4/A -49.262
Memory Used: 1.979 Mb
Reason for exit: Successful completion
Semi-Empirical Program CPU Time : 000:00:00.3

SPARTAN '06 Properties Program: (PC/x86) Release 127
Use of molecular symmetry disabled
Molecular charge: 0
Spin multiplicity: 1
Electrons: 94

Cartesian Coordinates (Angstroms)

Atom		X	Y	Z
1 C	C1	-2.0432380	-0.0383068	0.1861534
2 N	N1	-2.5958197	0.6400054	-0.7548697
3 N	N2	-0.6841846	-0.1346382	0.4061343
4 H	H3	-0.4359118	-0.2599738	1.3814000
5 N	N3	-2.8900997	-0.7408496	1.0647821
6 H	H5	-2.5396008	-1.6507829	1.3483341
7 C	C2	-4.0402060	0.4083339	-0.6272304
8 H	H1	-4.5265750	1.3238660	-0.2614005
9 H	H7	-4.4901891	0.1727803	-1.5986959
10 C	C3	-4.2036763	-0.7544768	0.3938223
11 H	H2	-5.0156221	-0.5896465	1.1088417
12 H	H8	-4.3820256	-1.7130173	-0.1146406
13 C	C4	0.2027100	0.7747178	-0.3207522
14 H	H4	0.0625910	1.8187380	-0.0033096
15 H	H6	-0.1039512	0.7321091	-1.3711216
16 C	C5	1.6537296	0.3730893	-0.1587170
17 C	C6	4.3575776	-0.3531491	0.0957607
18 C	C7	2.6207052	1.3266915	0.1769939
19 C	C8	2.0582617	-0.9541314	-0.3615697
20 C	C9	3.3988900	-1.3146926	-0.2344978
21 C	C10	3.9653338	0.9696017	0.3007097
22 H	H9	2.3173224	2.3582192	0.3432076
23 H	H10	1.3107487	-1.7013895	-0.6139147
24 H	H11	3.6982154	-2.3473071	-0.3957964
25 H	H12	4.7027130	1.7237309	0.5634123
26 H	H13	5.4024687	-0.6355603	0.1944898

Point Group = C1 Order = 1 Nsymop = 1

1-2 Energy and Cartesian Coordinates of the Optimized Geometries for 2

SPARTAN 'O6 Mechanics Program: (PC/x86) Release 127

Reading coordinates from previous archive
Adjusted 2 (out of 87) low frequency modes

Reason for exit: Successful completion
Mechanics CPU Time : 000:00:00.3

SPARTAN 'O6 Quantum Mechanics Program: (PC/x86) Release 127v3

Job type: Geometry optimization.
Method: RB3LYP
Basis set: 6-31G**
Number of shells: 103
Number of basis functions: 305

SCF model:
A restricted hybrid HF-DFT SCF calculation will be
performed using Pulay DIIS + Geometric Direct Minimization

Optimization:

Step	Energy	Max Grad.	Max Dist.
1	-741.6905708	0.161711	0.180353
2	-741.7319297	0.048534	0.108656
3	-741.7393401	0.019891	0.076428
4	-741.7413494	0.009815	0.030571
5	-741.7418837	0.003525	0.023325
6	-741.7420708	0.002679	0.070197
7	-741.7423428	0.001936	0.049497
8	-741.7424154	0.001289	0.003240
9	-741.7424200	0.001858	0.093416
10	-741.7425267	0.002556	0.097466
11	-741.7426595	0.006031	0.088921
12	-741.7427645	0.006694	0.075331
13	-741.7426961	0.006473	0.114015
14	-741.7430483	0.003961	0.121087

15	-741.7433303	0.005433	0.092337
16	-741.7434443	0.007786	0.098575
17	-741.7435409	0.008412	0.083895
18	-741.7435574	0.009820	0.072062
19	-741.7437774	0.005432	0.071782
20	-741.7438006	0.003554	0.040655
21	-741.7438818	0.002412	0.059684
22	-741.7439141	0.002400	0.071173
23	-741.7439745	0.003031	0.093374
24	-741.7440538	0.002216	0.080203
25	-741.7440908	0.001814	0.057559
26	-741.7441189	0.001866	0.074472
27	-741.7441623	0.001676	0.062714
28	-741.7441867	0.001693	0.080751
29	-741.7442228	0.002071	0.078633
30	-741.7442460	0.001915	0.089619
31	-741.7442776	0.001676	0.062501
32	-741.7443164	0.001886	0.073353
33	-741.7443594	0.001435	0.107550
34	-741.7444030	0.001067	0.107251
35	-741.7444491	0.001665	0.071698
36	-741.7444994	0.001367	0.102746
37	-741.7445298	0.002797	0.078080
38	-741.7445497	0.002372	0.109335
39	-741.7445730	0.002336	0.040476
40	-741.7445686	0.002331	0.047031
41	-741.7446060	0.001458	0.055677
42	-741.7446129	0.001855	0.034857
43	-741.7446313	0.000969	0.029833
44	-741.7446391	0.000691	0.038300
45	-741.7446464	0.000423	0.044905
46	-741.7446497	0.000820	0.025251
47	-741.7446517	0.000296	0.014202
48	-741.7446520	0.000235	0.012981

Program Wall Time: 8:37:45.0

Reason for exit: Successful completion

Quantum Calculation CPU Time : 006:51:21.5

SPARTAN 'O6 Semi-Empirical Program: (PC/x86) Release 127

Semi-empirical Property Calculation

M001

Guess from Archive

Energy Due to Solvation

Solvation Energy SM5.4/A -120.041

Memory Used: 2.866 Mb

Reason for exit: Successful completion

Semi-Empirical Program CPU Time : 000:00:00.5

SPARTAN '06 Properties Program: (PC/x86) Release 127

Use of molecular symmetry disabled

Molecular charge: 0

Spin multiplicity: 1

Electrons: 116

Cartesian Coordinates (Angstroms)

Atom		X	Y	Z
1 C	C1	1.4873019	-0.6531623	0.1618736
2 N	N1	2.4291976	0.2912939	0.0832539
3 N	N2	1.9373201	-1.8602002	-0.3038126
4 C	C2	3.4025329	-1.7736008	-0.4665329
5 H	H2	3.9140033	-2.1986929	0.4061983
6 H	H7	3.7273608	-2.3099044	-1.3605559
7 C	C3	3.6265137	-0.2492950	-0.5658293
8 H	H3	4.5283682	0.0897226	-0.0561470
9 H	H8	3.6748981	0.0959029	-1.6035654
10 N	N3	0.2680008	-0.4052223	0.6186230
11 H	H14	0.1815725	0.6199892	0.8171897
12 C	C11	-0.8655167	-1.3126955	0.5283020
13 H	H1	-0.6631981	-2.0453578	-0.2650796

14 H	H16	-0.9826224	-1.8775204	1.4631715
15 H	H17	1.5341874	-2.7059943	0.0739657
16 C	C4	-2.1643125	-0.5865519	0.2193385
17 C	C5	-4.6072160	0.6479467	-0.3971399
18 C	C6	-2.1870350	0.6188214	-0.4907480
19 C	C7	-3.3744058	-1.1623760	0.6237119
20 C	C8	-4.5893478	-0.5530292	0.3137441
21 C	C9	-3.4043687	1.2325024	-0.7924550
22 H	H5	-1.2585034	1.0962292	-0.7867109
23 H	H6	-3.3659545	-2.0919230	1.1880849
24 H	H9	-5.5196526	-1.0098944	0.6371044
25 H	H10	-3.4071941	2.1730670	-1.3344165
26 H	H11	-5.5521853	1.1290810	-0.6321654
27 C	C10	2.1590475	1.8136940	0.1596601
28 O	O1	3.1181422	2.4682396	-0.2149289
29 O	O2	1.0123341	2.0693388	0.5893191

Point Group = C1 Order = 1 Nsymop = 1

1-3 Energy and Cartesian Coordinates of the Optimized Geometries for 3

SPARTAN 'O6 Mechanics Program: (PC/x86) Release 127

Reading coordinates from previous archive
Adjusted 4 (out of 96) low frequency modes

Reason for exit: Successful completion
Mechanics CPU Time : 000:00:00.2

SPARTAN 'O6 Quantum Mechanics Program: (PC/x86) Release 127v3

Job type: Geometry optimization.
Method: RB3LYP
Basis set: 6-31G(D)
Number of shells: 98
Number of basis functions: 285

SCF model:
A restricted hybrid HF-DFT SCF calculation will be
performed using Pulay DIIS + Geometric Direct Minimization

Optimization:

Step	Energy	Max Grad.	Max Dist.
1	-818.1530672	0.041575	0.115866
2	-818.1652199	0.009168	0.128149
3	-818.1669486	0.004561	0.168157
4	-818.1676447	0.002078	0.163519
5	-818.1681501	0.002822	0.142481
6	-818.1685656	0.002628	0.192636
7	-818.1688968	0.002500	0.208203
8	-818.1692038	0.001713	0.205202
9	-818.1694676	0.001495	0.003240
10	-818.1694722	0.001487	0.162469
11	-818.1696671	0.000826	0.052926
12	-818.1696915	0.000569	0.020297
13	-818.1696994	0.000403	0.020242
14	-818.1697051	0.000265	0.005045

15	-818.1697083	0.000180	0.008785
16	-818.1697109	0.000093	0.006951
17	-818.1697120	0.000123	0.007275
18	-818.1697130	0.000081	0.008597

Program Wall Time: 1:22:40.0

Reason for exit: Successful completion
Quantum Calculation CPU Time : 001:21:43.0

SPARTAN 'O6 Semi-Empirical Program: (PC/x86) Release 127
Semi-empirical Property Calculation

M001

Guess from Archive

Energy Due to Solvation
Solvation Energy SM5.4/A -115.671
Memory Used: 3.493 Mb
Reason for exit: Successful completion
Semi-Empirical Program CPU Time : 000:00:00.4

SPARTAN '06 Properties Program: (PC/x86) Release 127
Use of molecular symmetry disabled
Molecular charge: 0
Spin multiplicity: 1
Electrons: 126

Cartesian Coordinates (Angstroms)

Atom		X	Y	Z
1 C	C1	3.5968575	1.1854565	0.1506869
2 O	O1	4.7107427	1.9743559	0.3041840
3 H	H1	4.3946598	2.8799882	0.1507422
4 O	O2	3.7885871	-0.0430397	0.2989908
5 O	O3	2.5208248	1.8016550	-0.1197117

6 N	N1	0.4105717	0.2969361	-0.6084122
7 C	C2	0.5225649	-0.9888203	-0.3162836
8 N	N2	1.5992675	-1.5283711	0.2557944
9 N	N3	-0.4351573	-1.9379911	-0.5826687
10 H	H7	-1.4031036	-1.6380996	-0.5548798
11 C	C3	1.4688064	-2.9827103	0.2876755
12 H	H5	2.0220110	-3.4437176	-0.5419005
13 H	H11	1.8454581	-3.3971351	1.2262924
14 C	C4	-0.0548038	-3.1740719	0.1225867
15 H	H9	-0.3107568	-4.0580446	-0.4663762
16 H	H12	-0.5560052	-3.2396478	1.0979244
17 C	C5	-0.7463335	0.8594532	-1.2944740
18 H	H4	-0.9071801	0.3456723	-2.2523027
19 H	H6	-0.4724974	1.8925291	-1.5328613
20 C	C6	-2.0341918	0.8346105	-0.4823695
21 C	C7	-4.4226939	0.8231442	0.9951989
22 C	C8	-3.2314569	0.4034700	-1.0664587
23 C	C9	-2.0451719	1.2583811	0.8546596
24 C	C10	-3.2309348	1.2529172	1.5869207
25 C	C11	-4.4215612	0.3987874	-0.3330699
26 H	H8	-3.2352458	0.0770734	-2.1048167
27 H	H10	-1.1178265	1.5872891	1.3162500
28 H	H13	-3.2269618	1.5860141	2.6213528
29 H	H14	-5.3430239	0.0620743	-0.8004871
30 H	H15	-5.3455618	0.8194312	1.5687463
31 H	H17	2.5187193	-0.9792196	0.2951476
32 H	H19	1.2678770	0.9150694	-0.4100233

Point Group = C1 Order = 1 Nsymop = 1

2-1. **^{1}H NMR spectra of (a) 1 observed in DMSO-d_6, (b) 3' prepared with 2 and D_2O observed in D_2O, and (c) 3 prepared with 1 under wet condition observed in D_2O.**

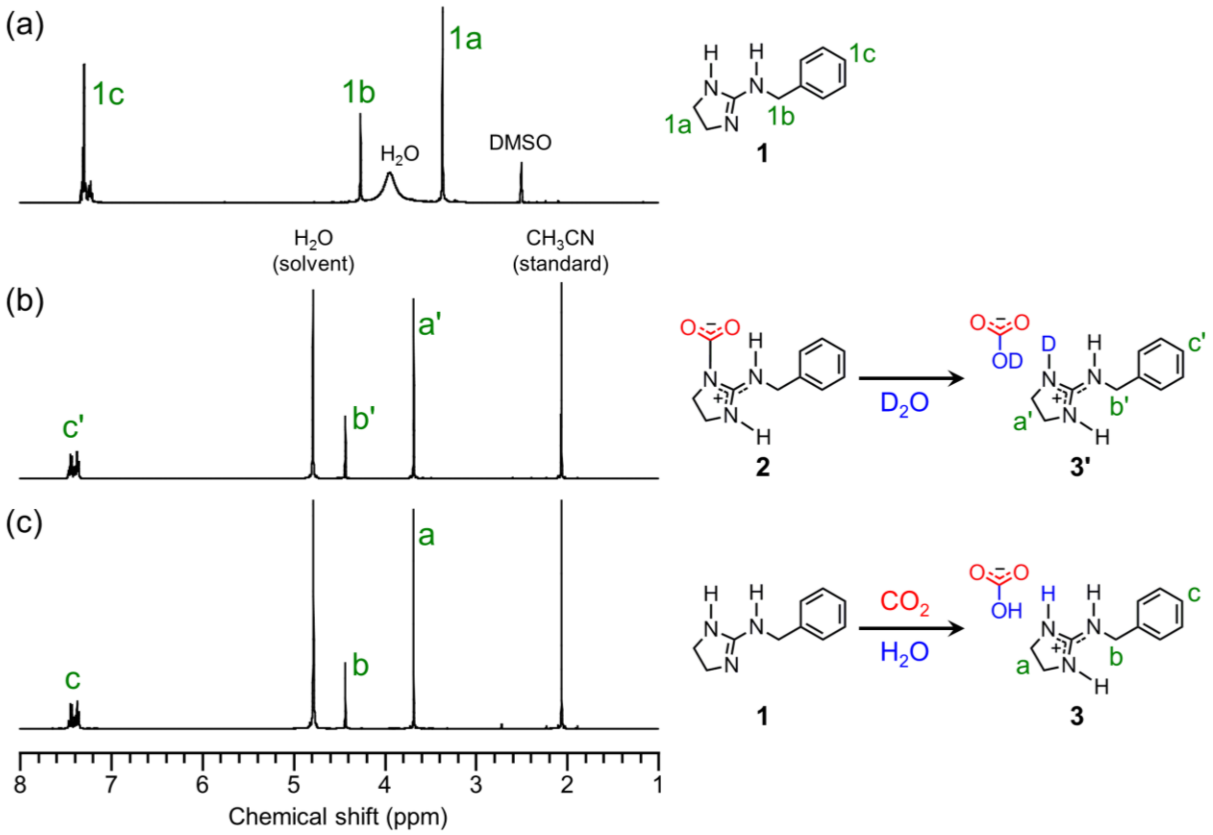

2-2. ^{13}C NMR spectra of (a) 1 observed in DMSO-d_6, (b) 3' prepared with 2 and D_2O observed in D_2O, and (c) 3 prepared with 1 under wet condition observed in D_2O.

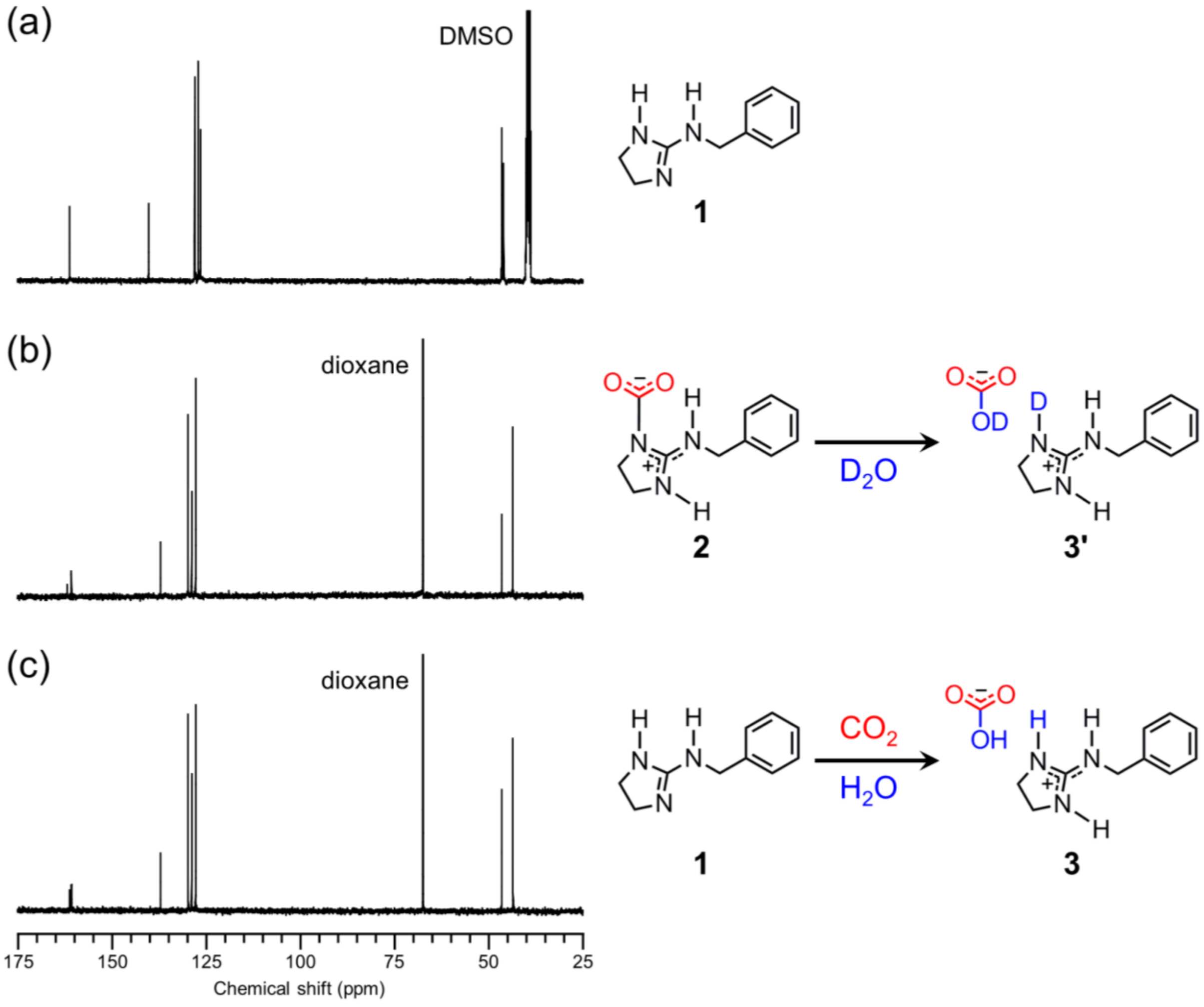

2-3. FTIR-ATR spectrum of 1

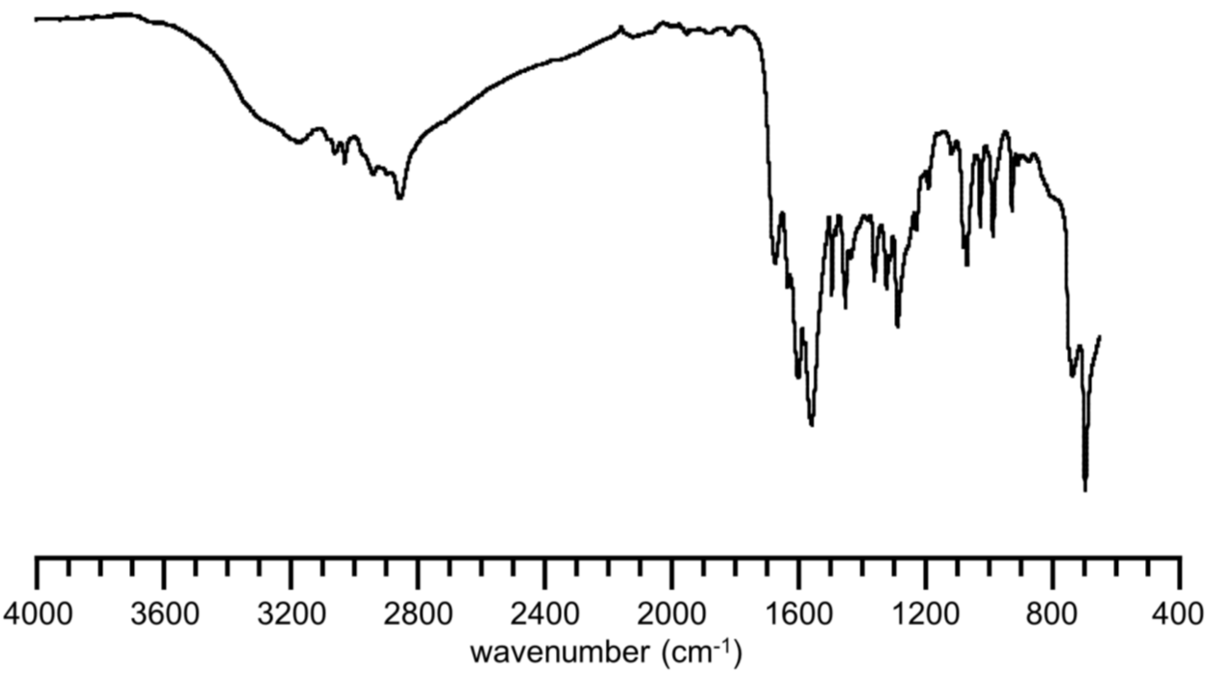

2-4. (a) FTIR-ATR and (b) ^{13}C-CPMAS NMR spectra of 2

(a) FTIR-ATR spectrum

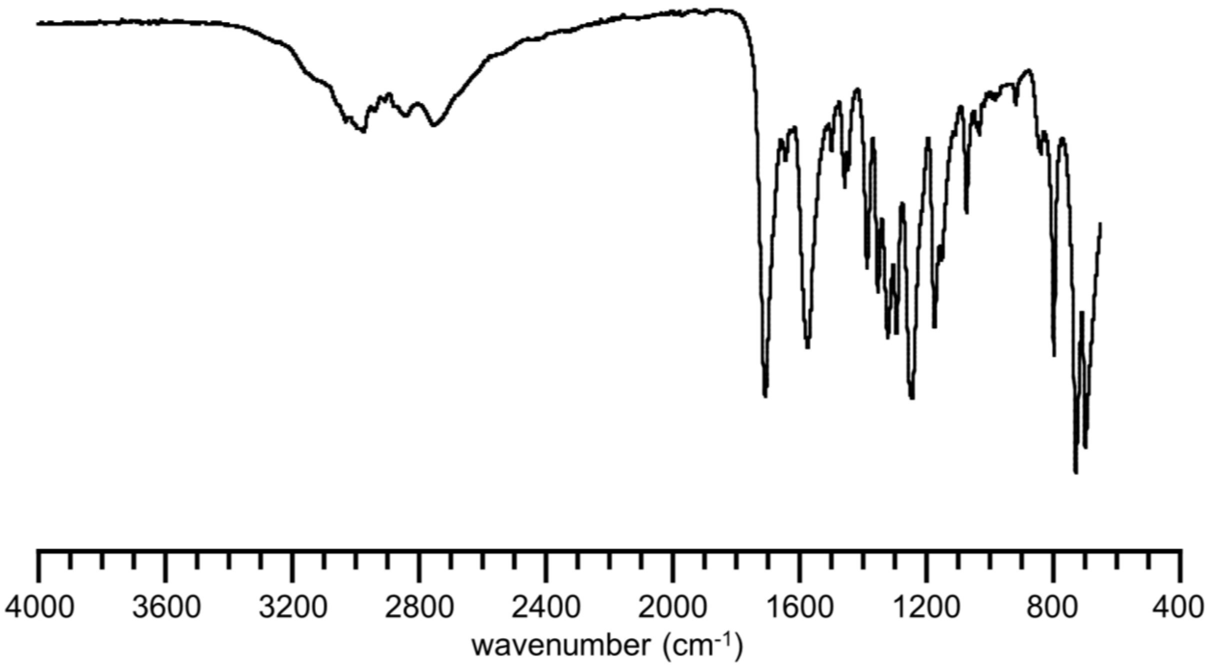

(b) ^{13}C-CPMAS NMR

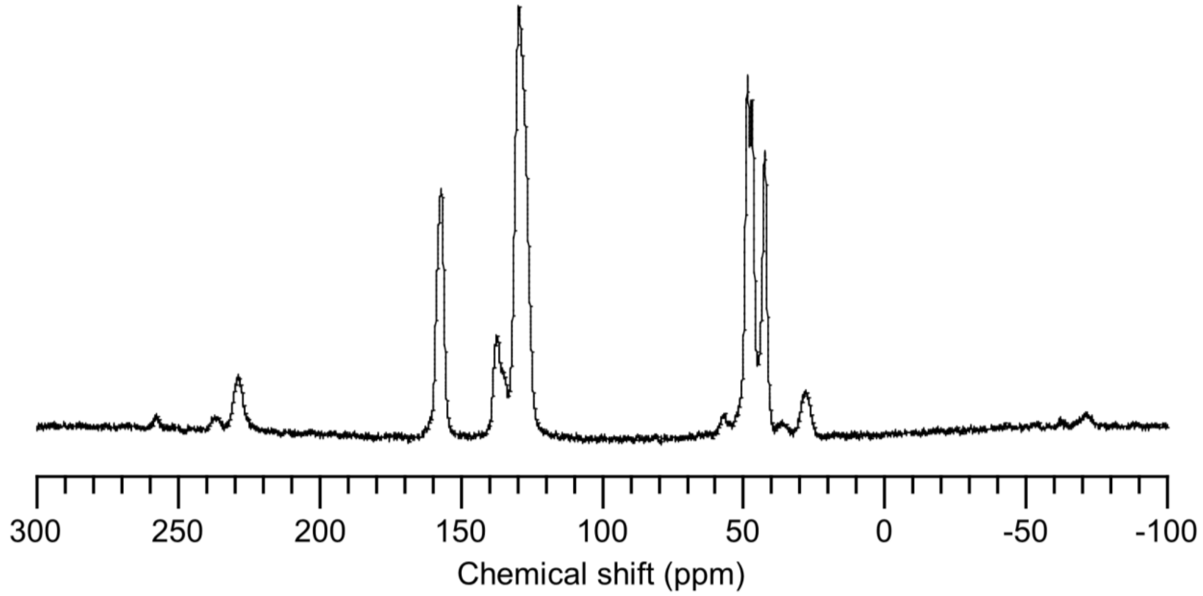

2-5. (a) FTIR-ATR and (b) ^{13}C-CPMAS NMR spectra of 3

(a) FTIR-ATR spectrum

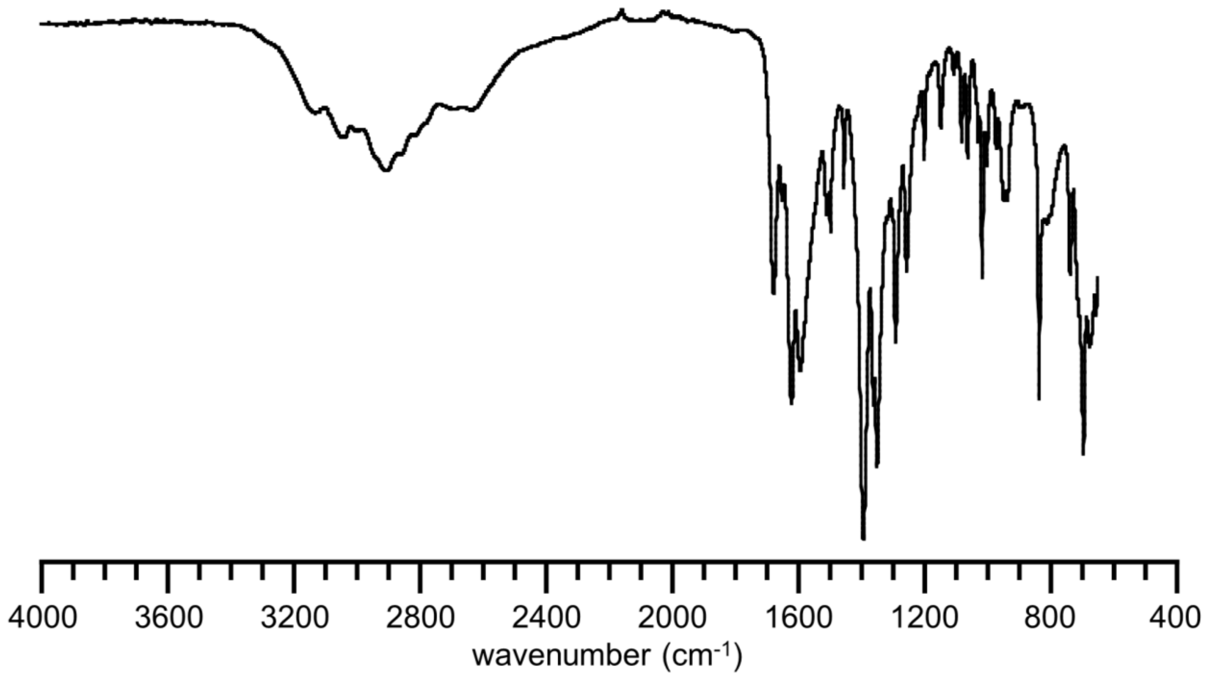

(b) ^{13}C-CPMAS NMR

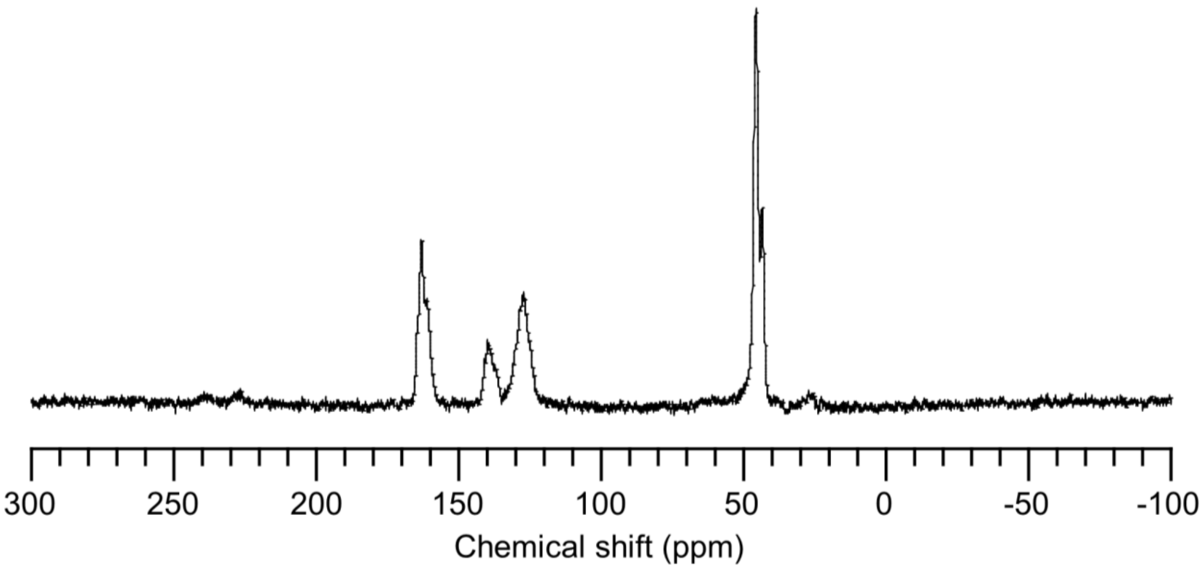

Supplement: File 1 — DFT computational results, FTIR-ATR, and NMR spectra of 1, 2 and 3. [file Beilstein_J_Org_Chem-14-2204-s001.pdf]
